# Usage of Neural Network to Predict Aluminium Oxide Layer Thickness

**DOI:** 10.1155/2015/253568

**Published:** 2015-04-02

**Authors:** Peter Michal, Alena Vagaská, Miroslav Gombár, Ján Kmec, Emil Spišák, Daniel Kučerka

**Affiliations:** ^1^Department of Mathematics, Informatics and Cybernetics, Faculty of Manufacturing Technologies with a Seat in Prešov, Technical University of Košice, Bayerova 1, 080 01 Prešov, Slovakia; ^2^Department of Mechanical Engineering, Institute of Technology and Businesses in České Budějovice, Okružní 10, 37001 České Budějovice, Czech Republic; ^3^Department of Technologies and Materials, Faculty of Mechanical Engineering, Technical University of Košice, Mäsiarska 74, 042 00 Košice, Slovakia

## Abstract

This paper shows an influence of chemical composition of used electrolyte, such as amount of sulphuric acid in electrolyte, amount of aluminium cations in electrolyte and amount of oxalic acid in electrolyte, and operating parameters of process of anodic oxidation of aluminium such as the temperature of electrolyte, anodizing time, and voltage applied during anodizing process. The paper shows the influence of those parameters on the resulting thickness of aluminium oxide layer. The impact of these variables is shown by using central composite design of experiment for six factors (amount of sulphuric acid, amount of oxalic acid, amount of aluminium cations, electrolyte temperature, anodizing time, and applied voltage) and by usage of the cubic neural unit with Levenberg-Marquardt algorithm during the results evaluation. The paper also deals with current densities of 1 A·dm^−2^ and 3 A·dm^−2^ for creating aluminium oxide layer.

## 1. Introduction

Pure aluminium and its alloys, such as weight-saving materials, play an increasingly important role of technical, technological, and economic terms [1] in the aerospace and automotive industries [2], where lightweight and rigid structure are preferred [3]. Aluminium alloys are also used to prevent or reduce damage in many engineering structures and components [4]. Anodic aluminium oxide (AAO) coating has recently attracted the scientists' attention because of its self-organizing nature of vertical (cylindrical) pores in the form of hexagonal arrays, which provides a controlled and narrow distribution of pore diameters and interpore distances in addition to the possibility of forming the pores with extremely high aspect ratio [5]. Anodizing is one of the most important processes in corrosion protection and colour finishes for aluminium [6]. Anodizing of aluminium surfaces is carried out in a wide variety of plants for numerous uses in industries. It is an effective process applied to producing decorative and protective films on articles made from aluminium [7]. Anodic oxidation is a most frequently used but also least explored method of surface treatment of aluminium profiles in terms of corrosion resistance increase [8]. With the oxidation of aluminium, when forming the electrolyte, the most frequently used are sulphuric acid and oxalic acid, alternatively a combination of them, because of their environmental friendliness [9, 10]. The mechanism of an oxide layer formation when using sulphuric acid solution has been examined by Tsangaraki-Kaplanogloua et al. [11], Patermarakis [12], and Aerts et al. [13], who managed to design a mathematical model of local turbulences in the electrolyte and examine their influence on the geometrical dimensions of the pores. Aerts et al. were also dealing with the temperature effect on the growth of the oxide layer and the layer porosity [5] of 99.50% aluminium using the electrolyte comprising sulphuric acid based on which it followed that the structure of the layer, the layer porosity, and its thickness and hardness are not so much under the influence of the temperature of the electrolyte compared to that of the electrode.

## 2. Experimental

Alloy EN AW 1050-H24 with dimensions 101 × 70 × 1 mm was used for specimens. Each applied specimen was degreased in a 38.00% solution of NaOH at 55 to 60°C for 2 minutes and stained in a 40.00% solution of NaOH at the temperature 45°–50°C for 0.50 min. Consequently, the specimen was immersed in a nitric acid bath (4.00% HNO_3_) at the temperature from 18 to 24°C for 1.00 min. After operations of degreasing and staining, the sample was rinsed with distilled water.

Electrolyte for each anodizing sample was made from sulphuric acid, oxalic acid, and free aluminium oxide (added like powdered aluminium oxide).  Table 1 shows transfers of factors between nature scale and coded scale. Coded scale is used to prevent influence of the absolute value of the studied factors in evaluating the results of the experiment.

## 3. Problem Solution

In 1943 McCulloch and Pitts laid the foundations of the theory of neural networks. Since then the neural network has become an important tool in the field of artificial intelligence, simulation, control, and optimization of processes and in the field of natural and social sciences [14–16]. A higher-order neural unit (HONU), especially the 3rd order HONU based on the iterative Levenberg-Marquardt (LM) algorithm [17–19], was used to determine the influence of input factors on the thickness of the final AAO layer. This algorithm is often used for training technique of the neural unit [6, 20]. It is a process of updating individual weights in a predetermined number of steps to achieve a minimum difference between the actual and calculated values of observed variable [21–23]. The equation describing the investigated model is the characteristic equation of given type of neural unit (1st order HONU, 2nd order HONU, and a 3rd order HONU) for observed factors *x*_1_, *x*_2_, *x*_3_, *x*_4_, *x*_5_, and *x*_6_. In opposition to classical statistical methods for evaluating experimentally obtained data, the usage of one neuron unit only allows us to achieve higher accuracy and reliability of created prediction model. On the other hand, it is not possible to obtain such high accuracy and reliability in comparison to using a complex neural network but we can exclude neural network as a “black box” between input and output [24, 25]. This is very important in case of describing an examined technological process. If we have a “black box” between an input and output we are not able to control what is happening inside. In this case it is very probable that we are considering factors which are not relevant but they are deforming the resulting model. It is possible to identify irrelevant factors under certain conditions, but it is important to have very practical experience with examined process.

## 4. Results and Discussions

After the learning process of neuron unit is done, we get a prediction model that describes the thickness of AAO layer. The final thickness of oxide layer, *α*, is preliminary thickness of oxide layer which is expressed in mm·10^−3^.  Table 2 shows significant statistical indicator for compiled prediction models of surface AAO layer thickness for surface current densities 1 A·dm^−2^ and 3 A·dm^−2^. Those indicators are sum of square errors, “SSE,” root mean square error, “RMSE,” correlation coefficient, “*R*,” coefficient of determination, “*R*,” standard deviation of errors, “se,” variation of errors, “s^2^e,” and biggest error of prediction, “maxe.”

Table 3 shows thickness differences (Δ*h*) between measured layer thicknesses for current density 1 A·dm^−2^ (*h*_1A_) and measured layer thicknesses for current density 3 A·dm^−2^ (*h*_3A_). We can see in Table 3 the resulting differences are mostly in range from −1 *μ*m to 1 *μ*m. Based on this the general statement that the current density is a factor which has significant impact on resulting layer thickness of anodic aluminium oxide is not always true. There are some differences between layer thicknesses for different current densities, especially in case of a higher concentration of sulphuric acid in electrolyte or in case of higher voltage applied, but it is difficult to determine if the oxide layer thickness will be thinner or thicker in the area of lower surface current density. To sum up it is better to claim that the current density has significant impact to internal structure of oxide layer.

**Figure 1:**
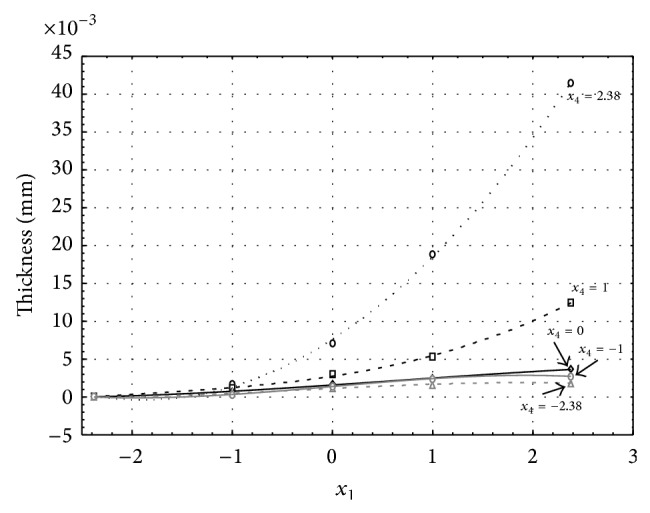
Influence of factors *x*_1_ and *x*_4_ on AAO layer thickness at current density 1 A·dm^−2^ and factor *x*_5_ which is set to level −2.38.

**Figure 2:**
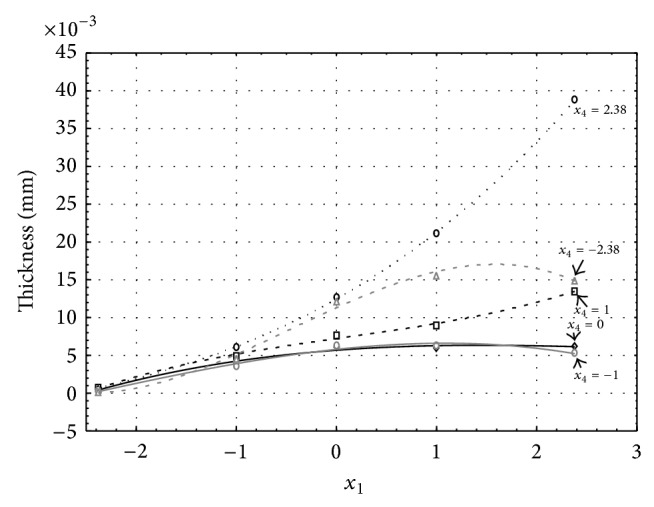
Influence of factors *x*_1_ and *x*_4_ on AAO layer thickness at current density of 1 A·dm^−2^ and factor *x*_5_ which is set to level −1.

**Figure 3:**
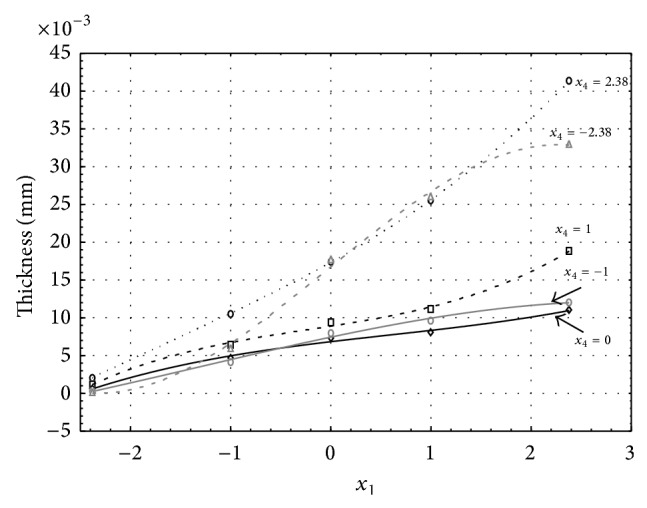
Influence of factors *x*_1_ and *x*_4_ on AAO layer thickness at current density of 1 A·dm^−2^ and factor *x*_5_ which is set to level 0.

**Figure 4:**
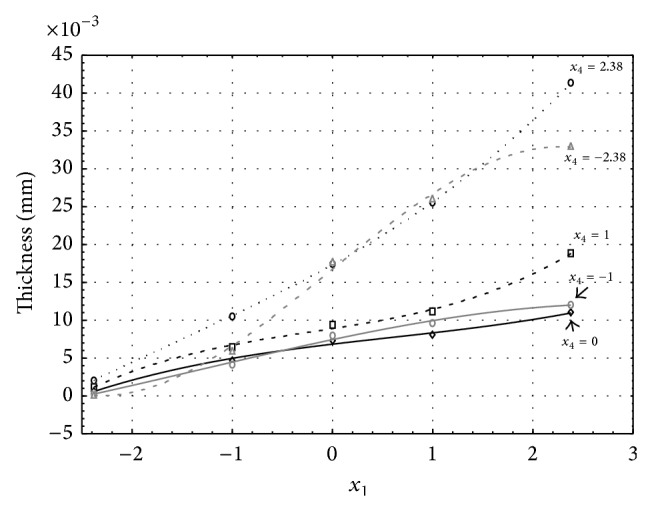
Influence of factors *x*_1_ and *x*_4_ on AAO layer thickness at current density of 1 A·dm^−2^ and factor *x*_5_ which is set to level 1.

**Figure 5:**
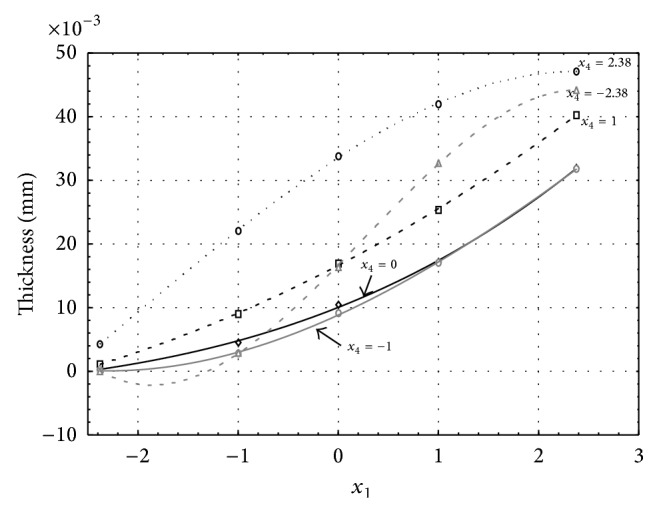
Influence of factors *x*_1_ and *x*_4_ on AAO layer thickness at current density of 1 A·dm^−2^ and factor *x*_5_ which is set to level 2.38.

Figures 1, 2, 3, 4, and 5 show the influence of factors *x*_1_ (concentration of sulphuric acid in the electrolyte) and *x*_4_ (temperature of the electrolyte) on the thickness of aluminium oxide created on sample surface. These graphs also demonstrate influence of factor *x*_5_ (anodizing time) on the oxide thickness. The level of factor *x*_5_ is set to level “−2.38” (6.22 min) Figure 1, “−1” (20 min) Figure 2, “0” (30 min) Figure 3, “1” (40 min) Figure 4, and “2.38” (53.78 min) Figure 5. Aluminium oxide layer was created on the surface areas at 1.00 A·dm^−2^ of current density. Factors *x*_2_, *x*_3_, and *x*_6_ have zero factor level for all these graphs. Zero factor level for factor *x*_2_ is 11 g·L^−1^, for factor *x*_3_ it is 8.5 g·L^−1^, and for factor *x*_6_ it is 10 V.

From these graphical characteristics it can be surmised that the thickness of AAO layer is proportional to concentration of sulphuric acid in the electrolyte (factor *x*_1_). Thus we can state that with increasing amount of sulphuric acid in the electrolyte also rises an amount of dissociated ions. Increased ion amount in an electrolyte increases its conductivity. Oxygen, which is bound to a part of these ions, is used to create a layer of an aluminium oxide. Electrolyte temperature (factor *x*_4_) influences the speed of oxide layer creating and also the thickness of AAO layer. With increasing temperature also rises the speed of chemical reactions on metal-electrolyte interface. However, general claim that the thickness of AAO layer is proportional to electrolyte temperature is not true. This claim is true only in a specific case. It means that some other variables significantly influence the thickness of AAO layer, specifically, the time of oxidation (factor *x*_5_). If the concentration of sulphuric acid in electrolyte influences the amount of ions in electrolyte and if electrolyte temperature influences the speed of chemical reactions on a metal-electrolyte interface, then time of oxidation determinates time of chemical reactions not only between meal and electrolyte but also between electrolyte and already created oxide layer. Reactions between metal and electrolyte create new molecules of aluminium oxide on the surface of metal and thus contribute to the rise of oxide layer. However, reactions between oxide layer and electrolyte cause reduction in thickness of created oxide layer due to its dissolving in the solution. Thus with the increase in time of oxidation, the thickness of oxide layer decreases, due to increase in electrolyte temperature. After crossing a certain temperature threshold (factor level −1 for Figure 2, factor level 0 for Figures 3, 4, and 5), the resulting oxide layer thickness increases. Speed of creating of oxide layer is higher than speed of melting already created aluminium oxide.

**Figure 6:**
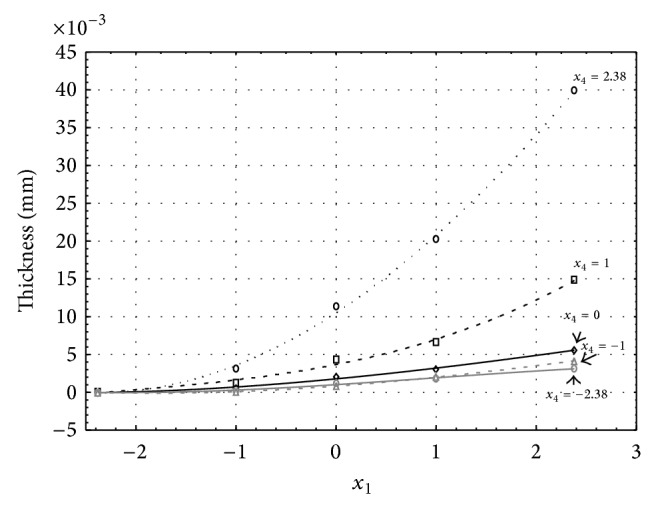
Influence of factors *x*_1_ and *x*_4_ on AAO layer thickness for current density 3 A·dm^−2^ and factor *x*_5_ which is set to level −2.38.

**Figure 7:**
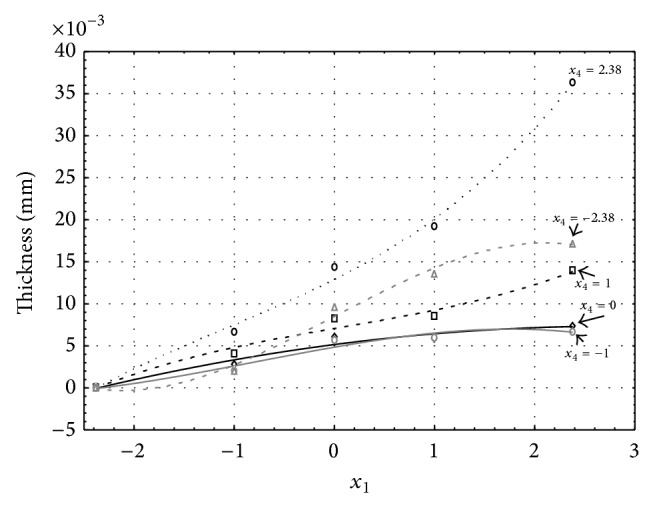
Influence of factors *x*_1_ and *x*_4_ on AAO layer thickness at current density of 3 A·dm^−2^ and factor *x*_5_ which is set to level −1.

**Figure 8:**
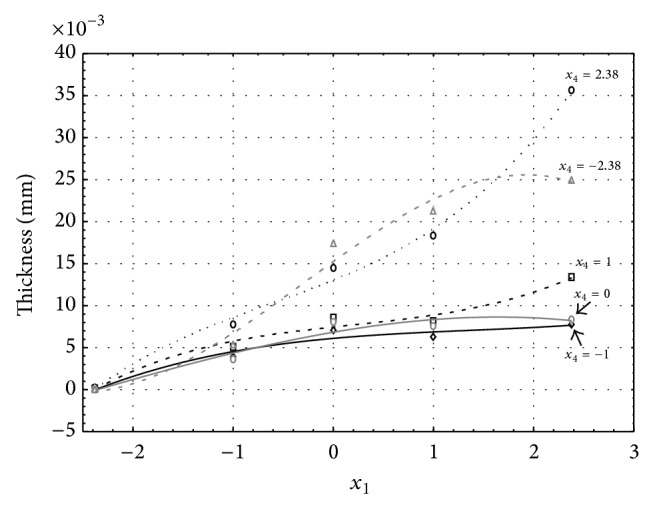
Influence of factors *x*_1_ and *x*_4_ on AAO layer thickness at current density of 3 A·dm^−2^ and factor *x*_5_ which is set to level 0.

**Figure 9:**
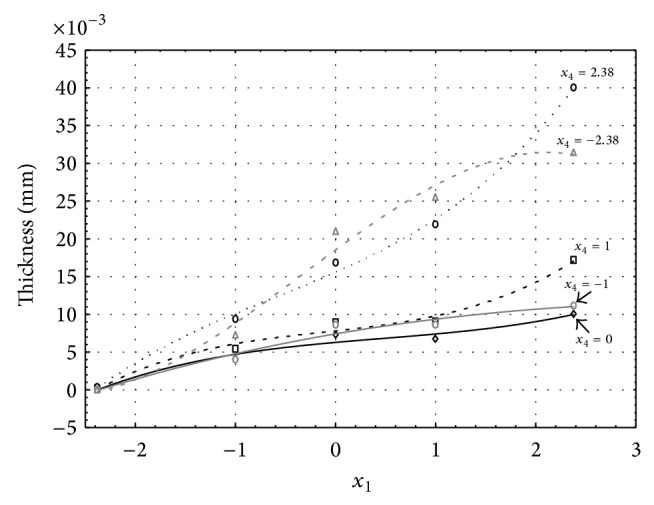
Influence of factors *x*_1_ and *x*_4_ on AAO layer thickness at current density of 3 A·dm^−2^ and factor *x*_5_ which is set to level 1.

**Figure 10:**
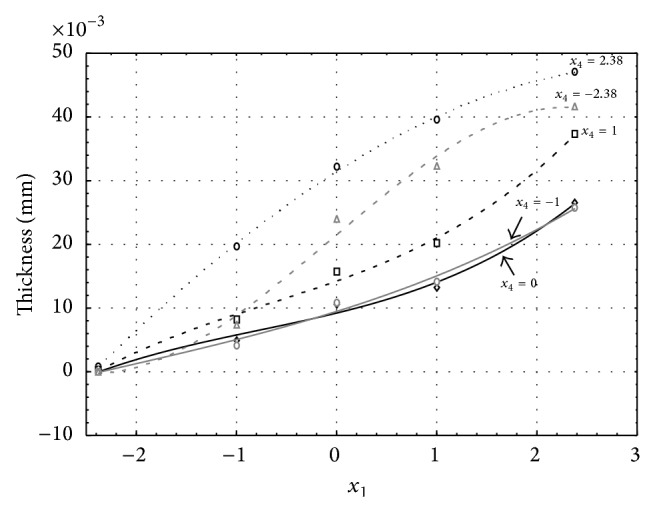
Influence of factors *x*_1_ and *x*_4_ on AAO layer thickness at current density of 3 A·dm^−2^ and factor *x*_5_ which is set to level 2.38.

Figures 6, 7, 8, 9, and 10 show influence of factors *x*_1_ (concentration of sulphuric acid in the electrolyte) and *x*_4_ (temperature of the electrolyte) on thickness of aluminium oxide created on sample surface. These graphs also demonstrate influence of factor *x*_5_ (anodizing time) on the oxide thickness. Level of factor *x*_5_ is set to level “−2.38” (6.22 min) Figure 6, “−1” (20 min) Figure 7, “0” (30 min) Figure 8, “1” (40 min) Figure 9, and “2.38” (53.78 min) Figure 10. Aluminium oxide layer was created on 3.00 A·dm^−2^ current density surface areas. Factors *x*_2_, *x*_3_, and *x*_6_ have zero factor level for all these pictures. Zero factor level for factor *x*_2_ is 11 g·L^−1^, for factor *x*_3_ it is 8.5 g·L^−1^, and for factor *x*_6_ it is 10 V. We can see that with increasing amount of sulphuric acid in electrolyte the thickness of a resulting aluminium oxide layer generally increases, too, as in case of surface current density 1 A·dm^−2^ (Figures 1, 2, 3, 4, and 5).

From comparison of thickness based on concentration of sulphuric acid in electrolyte, electrolyte temperature, and time of oxidation for current densities of 1 A·dm^−2^ and 3 A·dm^−2^ (Figures 1–10), it is evident that current density does not have a significant influence on the thickness of oxide layer if concentration of sulphuric acid is lower as at factor level 0. With its higher concentration, the thickness of oxide layer increases by approximately 5 mm·10^−3^ at current density of 3 A·dm^−2^.

**Figure 11:**
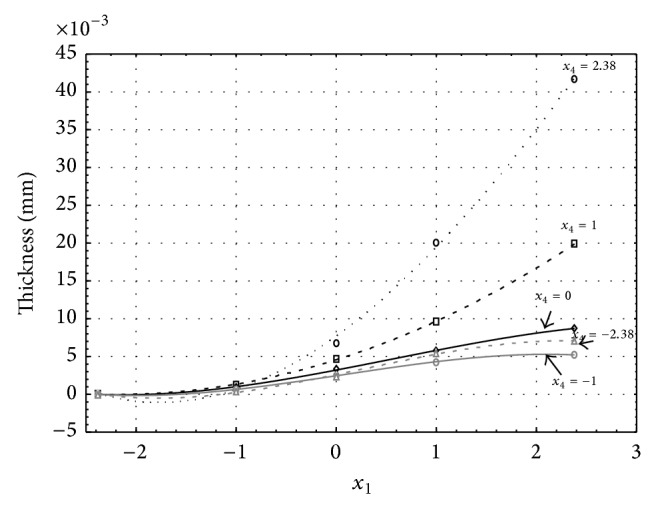
Influence of factors *x*_1_ and *x*_4_ on AAO layer thickness at current density of 1 A·dm^−2^ and factor *x*_6_ which is set to level −2.38.

Just as Figures 1 through 10 examine the relationship between the amount of sulphuric acid in electrolyte, electrolyte temperature, and oxidation time and thickness of oxide layer, Figures 11 through 20 show the influence of amount of sulphuric acid in electrolyte, electrolyte temperature, and voltage levels in relation to the thickness of the oxide layer. Results are shown for cases of current densities 1 A·dm^−2^ and 3 A·dm^−2^.

**Figure 12:**
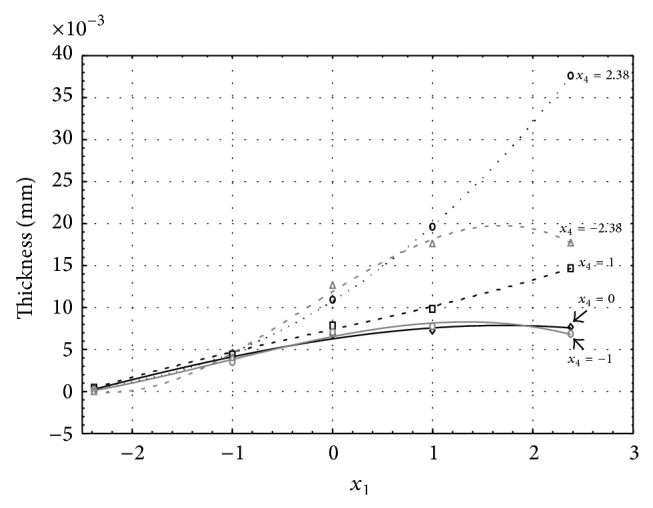
Influence of factors *x*_1_ and *x*_4_ on AAO layer thickness at current density of 1 A·dm^−2^ and factor *x*_6_ which is set to level −1.

**Figure 13:**
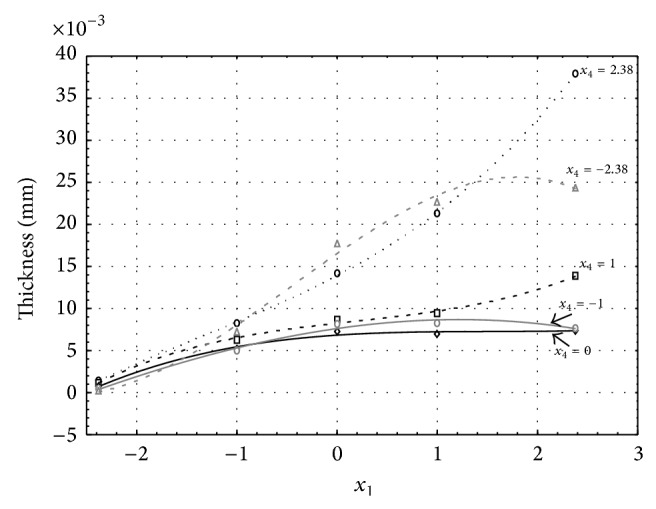
Influence of factors *x*_1_ and *x*_4_ on AAO layer thickness at current density of 1 A·dm^−2^ and factor *x*_6_ which is set to level 0.

**Figure 14:**
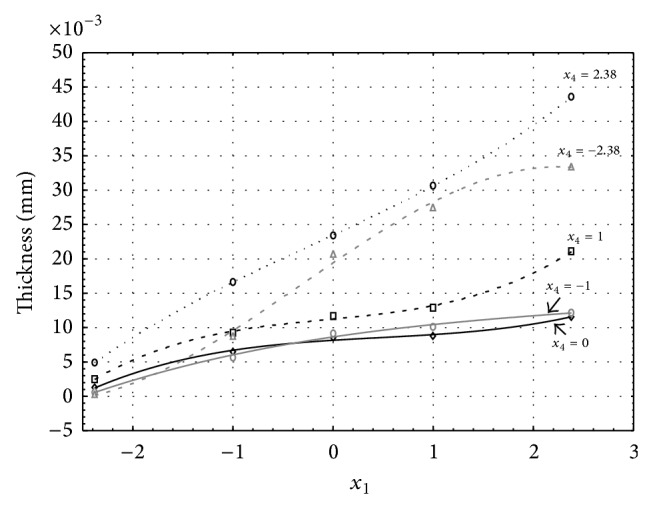
Influence of factors *x*_1_ and *x*_4_ on AAO layer thickness at current density of 1 A·dm^−2^ and factor *x*_6_ which is set to level 1.

**Figure 15:**
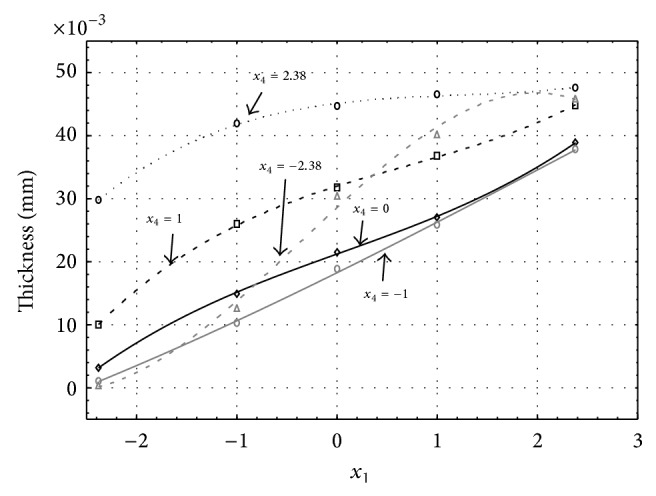
Influence of factors *x*_1_ and *x*_4_ on AAO layer thickness at current density of 1 A·dm^−2^ and factor *x*_6_ which is set to level 2.38.

Figures 11, 12, 13, 14, and 15 show the influence of factors *x*_1_ (concentration of sulphuric acid in the electrolyte) and *x*_4_ (temperature of the electrolyte) on the thickness of aluminium oxide created on sample surface. These graphs also demonstrate the influence of factor *x*_6_ (the size of an applied voltage) on the oxide thickness. Level of factor *x*_6_ is set to level “−2.38” (5.24 V) Figure 11, “−1” (8 V) Figure 12, “0” (10 V) Figure 13, “1” (12 V) Figure 14, and “2.38” (14.76 V) Figure 15. Aluminium oxide layer was created at 1.00 A·dm^−2^ current density surface areas. Factors *x*_2_, *x*_3_, and *x*_5_ have zero factor level for all these graphs. Zero factor level for factor *x*_2_ is 11 g·L^−1^, for factor *x*_3_ is 8.5 g·L^−1^, and for factor *x*_5_ is 30 min.

**Figure 16:**
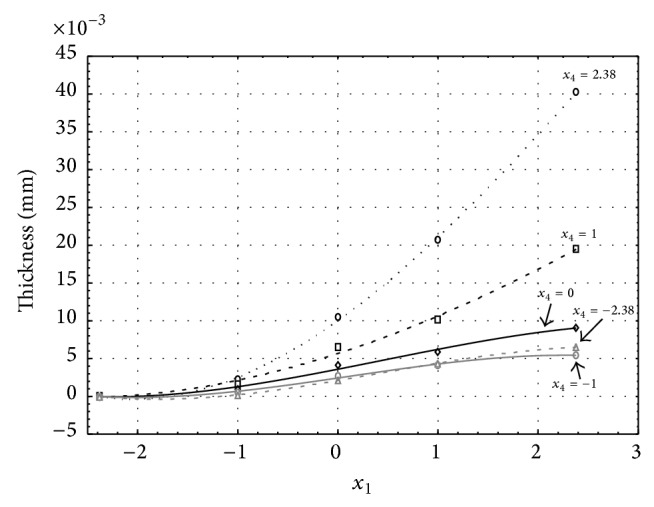
Influence of factors *x*_1_ and *x*_4_ on AAO layer thickness at current density 3 A·dm^−2^ and factor *x*_6_ which is set to level −2.38.

Connected voltage levels are proportional to the electric potential. Electric potential is proportional to electrodynamics forces. These electrodynamics forces determine the force with which are ions attracted to anode and cathode. If we increase voltage, electric potential on anode will also increase. Higher electric potential on anode will attract higher number of oxygen anions. Thus, the surface of aluminium sample will contain higher amount of oxygen anions and more molecules of aluminium oxide will be created on the surface of the sample. Through this, the thickness of AAO layer increases. It is possible to see this process in Figures 11 through 20, the same for current densities of 1 A·dm^−2^ (Figures 11 through 15) and 3 A·dm^−2^ (Figures 16 through 20), where the thickness of oxide layer increases faster with the increase of voltage.

**Figure 17:**
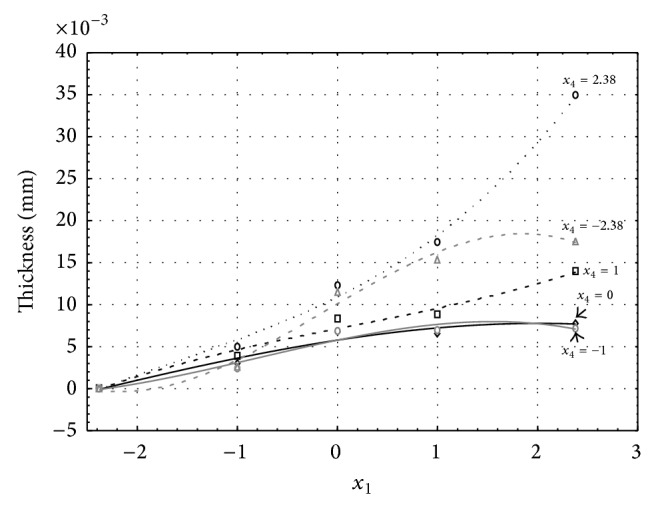
Influence of factors *x*_1_ and *x*_4_ on AAO layer thickness at current density of 3 A·dm^−2^ and factor *x*_6_ which is set to level −1.

**Figure 18:**
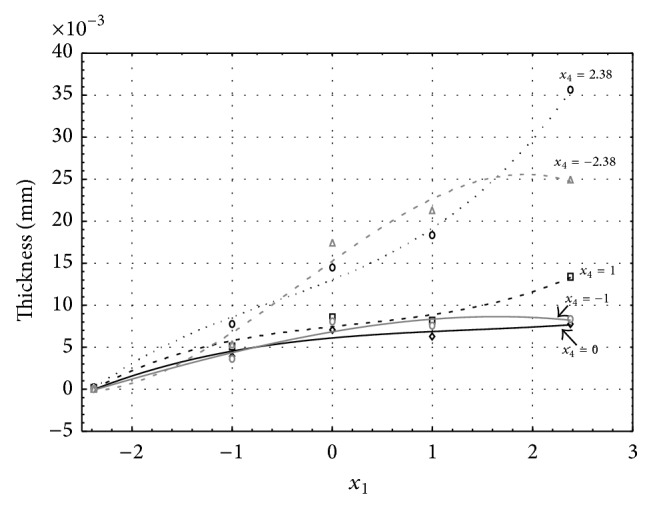
Influence of factors *x*_1_ and *x*_4_ on AAO layer thickness at current density of 3 A·dm^−2^ and factor *x*_6_ which is set to level 0.

**Figure 19:**
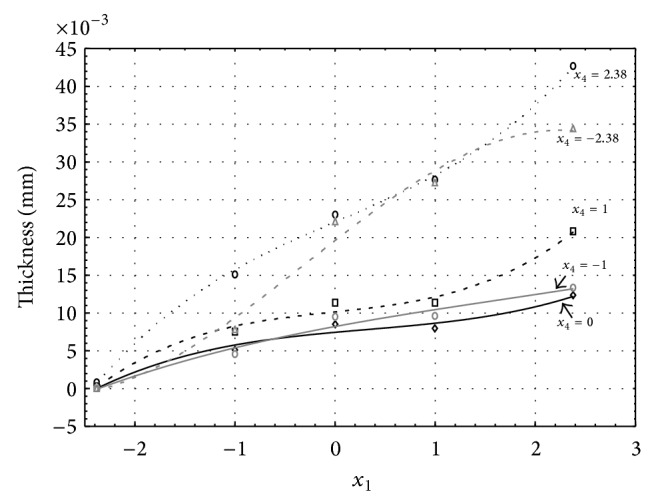
Influence of factors *x*_1_ and *x*_4_ on AAO layer thickness at current density of 3 A·dm^−2^ and factor *x*_6_ which is set to level 1.

**Figure 20:**
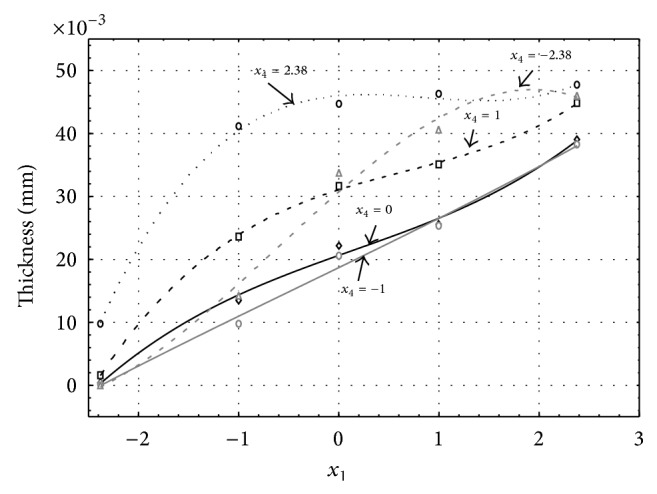
Influence of factors *x*_1_ and *x*_4_ on AAO layer thickness at current density of 3 A·dm^−2^ and factor *x*_6_ which is set to level 2.38.

Figures 16, 17, 18, 19, and 20 show the influence of factors *x*_1_ (concentration of sulphuric acid in the electrolyte) and *x*_4_ (temperature of the electrolyte) on the thickness of aluminium oxide created on sample surface. These graphs also demonstrate the influence of factor *x*_5_ (anodizing time) on the oxide thickness. Level of factor *x*_5_ is set to level “−2.38” (6.22 min) Figure 16, “−1” (20 min) Figure 17, “0” (30 min) Figure 18, “1” (40 min) Figure 19, and “2.38” (53.78 min) Figure 20. Aluminium oxide layer was created at 1.00 A·dm^−2^ current density surface areas. Factors *x*_2_, *x*_3_, and *x*_6_ have zero factor level for all these pictures. Zero factor level for factor *x*_2_ is 11 g·L^−1^, for factor *x*_3_ is 8.5 g·L^−1^, and for factor *x*_6_ is 10 V. By comparing the effects of input factors *x*_1_ (concentration of sulphuric acid in electrolyte), *x*_4_ (electrolyte temperature), and *x*_6_ (voltage level) at current density 1 A·dm^−2^ (Figures 11 through 15) and at current density 3 A·dm^−2^ (Figures 16 through 20) it is possible to surmise that levels of surface current density have no influence on the resulting thickness of oxide layer. Differences in thickness of AAO layer are minimal, as is the case with input factors *x*_1_ (concentration of sulphuric acid in electrolyte), *x*_4_ (electrolyte temperature), and *x*_5_ (time of oxidation) for current density 1 A·dm^−2^ (Figures 1–5) and at current density 3 A·dm^−2^ (Figures 6–10).

**Figure 21:**
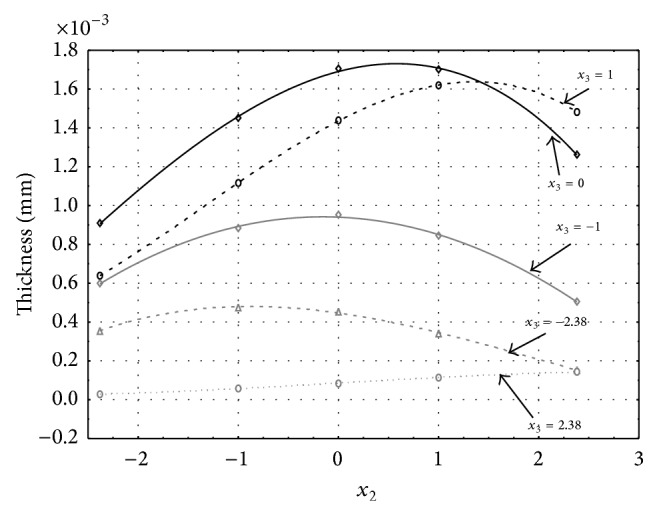
Influence of factors *x*_2_ and *x*_3_ on AAO layer thickness at current density of 1 A·dm^−2^ and factor *x*_5_ which is set to level −2.38.

**Figure 22:**
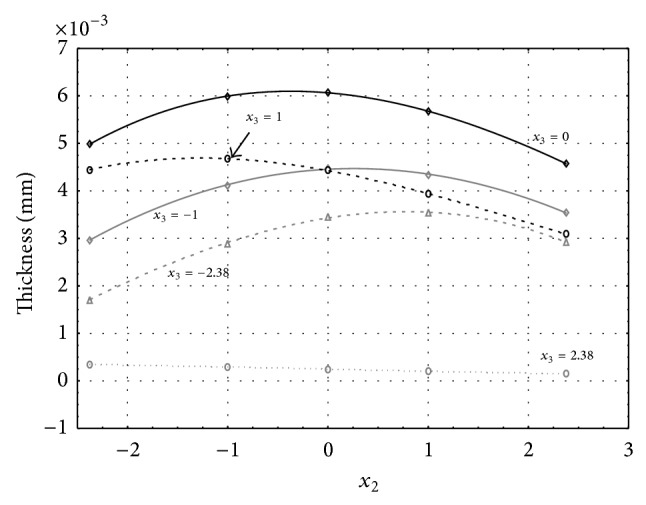
Influence of factors *x*_2_ and *x*_3_ on AAO layer thickness at current density of 1 A·dm^−2^ and factor *x*_5_ which is set to level −1.

**Figure 23:**
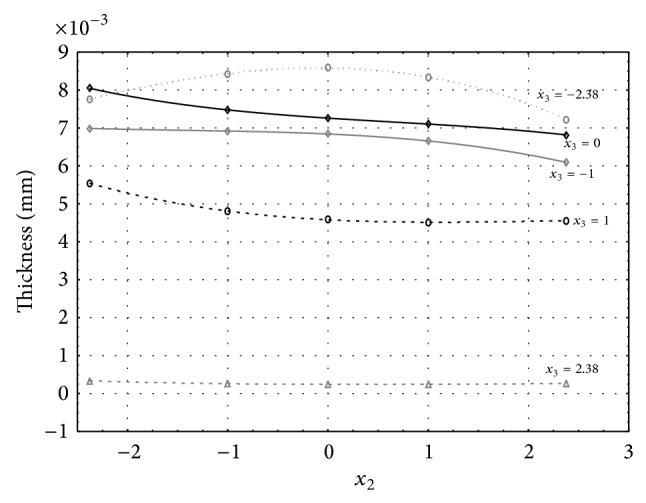
Influence of factors *x*_2_ and *x*_3_ on AAO layer thickness at current density of 1 A·dm^−2^ and factor *x*_5_ which is set to level 1.

**Figure 24:**
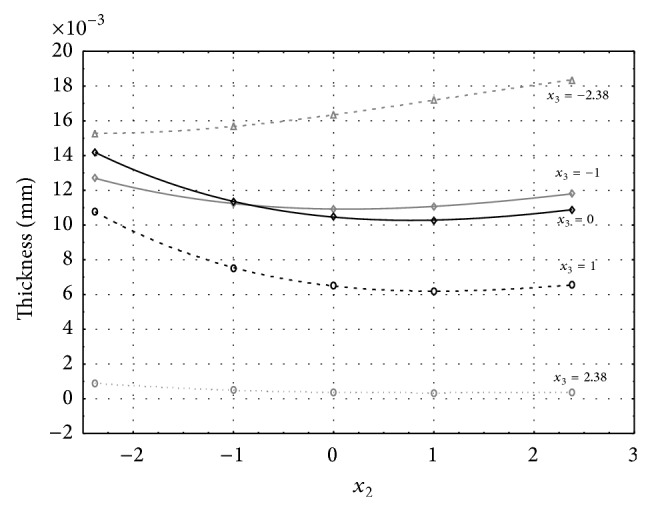
Influence of factors *x*_2_ and *x*_3_ on AAO layer thickness at current density of 1 A·dm^−2^ and factor *x*_5_ which is set to level 2.38.

**Figure 25:**
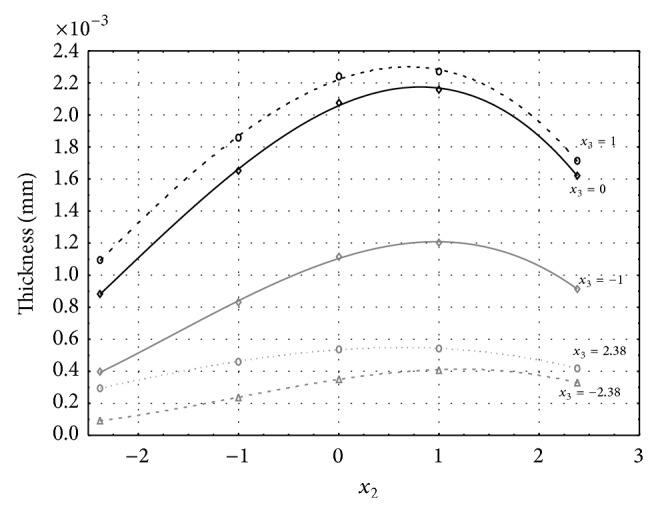
Influence of factors *x*_2_ and *x*_3_ on AAO layer thickness at current density of 3 A·dm^−2^ and factor *x*_5_ which is set to level −2.38.

**Figure 26:**
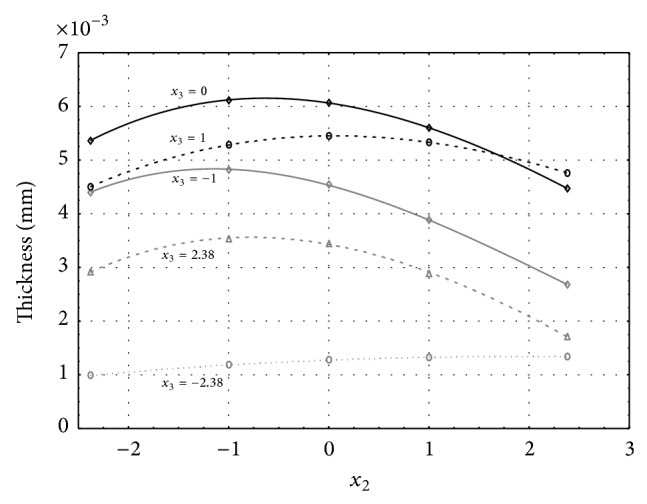
Influence of factors *x*_2_ and *x*_3_ on AAO layer thickness at current density of 3 A·dm^−2^ and factor *x*_5_ which is set to level −1.

**Figure 27:**
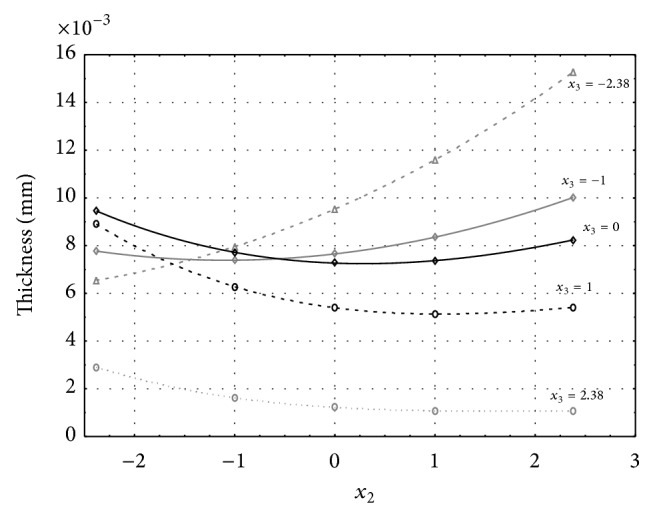
Influence of factors *x*_2_ and *x*_3_ on AAO layer thickness at current density of 3 A·dm^−2^ and factor *x*_5_ which is set to level 1.

**Figure 28:**
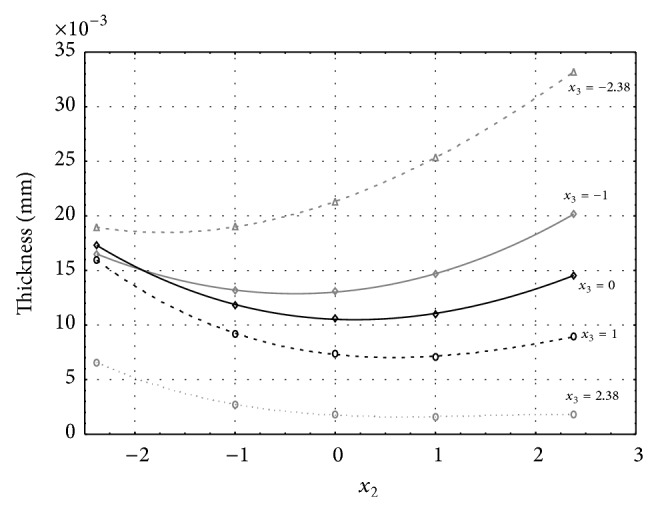
Influence of factors *x*_2_ and *x*_3_ on AAO layer thickness at current density of 3 A·dm^−2^ and factor *x*_5_ which is set to level 2.38.

Figures 21, 22, 23, and 24 show the influence of factors *x*_2_ (concentration of oxalic acid in the electrolyte) and *x*_3_ (concentration of aluminium cations in the electrolyte) on the thickness of aluminium oxide created on a sample surface. These graphs also demonstrate the influence of factor *x*_5_ (anodizing time) on the oxide thickness. The level of factor *x*_5_ is set to “−2.38” (6.22 min) Figure 21, “−1” (20 min) Figure 22, “1” (40 min) Figure 23, and “2.38” (53.78 min) Figure 24. Aluminium oxide layer was created at the current density surface area 1.00 A·dm^−2^. Figures 25, 26, 27, and 28 show the influence of factors *x*_2_ (concentration of oxalic acid in the electrolyte) and *x*_3_ (concentration of aluminium cations in the electrolyte) on the thickness of aluminium oxide created on sample surface. These graphs also demonstrate the influence of factor *x*_5_ (anodizing time) on the oxide thickness. The level of factor *x*_5_ is set to “−2.38” (6.22 min) Figure 25, “−1” (20 min) Figure 26, “1” (40 min) Figure 27, and “2.38” (53.78 min) Figure 28. Aluminium oxide layer was created at the current density surface areas of 3.00 A·dm^−2^. Factors *x*_1_, *x*_4_, and *x*_6_ have zero factor level for all of these pictures. Zero factor level for factor *x*_1_ is 200 g·L^−1^, for factor *x*_4_ is 22°C, and for factor *x*_6_ is 10 V.

By comparing the effects of input factors *x*_2_ (concentration of sulphuric acid in electrolyte), *x*_3_ (concentration of aluminium cations in electrolyte), and *x*_5_ (oxidation time) at current density 1 A·dm^−2^ (Figures 21 through 24) and at current density 3 A·dm^−2^ (Figures 25 through 28), it is possible to deduct that levels of surface current density have influence on the resulting thickness of oxide layer, as it is clearly shown especially in Figures 23 and 27 and in Figures 24 and 28.

## 5. Conclusion

As shown by the evaluation process of experimental results presented above, the use of 3rd order neural unit based on the iterative Levenberg-Marquardt (LM) optimization algorithm provides a wide range of options to investigate influence of input factors on the final AAO layer thickness. By using neural unit we can quickly and simply describe the behaviour of the monitored system. This neural unit allowed us to monitor the impact of input factors (concentration of sulphuric acid, electrolyte temperature, anodizing time, and applied voltage) on the final thickness of the AAO layer at surface current densities 1 A·dm^−2^ and 3 A·dm^−2^. Also by using the neural unit of 3rd order HONU, it was possible to describe the influence of input factors on the thickness of final AAO layer with confidence interval of 93.45% at surface current density 1 A·dm^−2^ and with confidence interval of 95.60% at surface current density 3 A·dm^−2^.

## Figures and Tables

**Table 1:** Table of transfers between natural scale and coded scale of examined factors.

Factor		Factor level				
Coded scale	Nature scale	−2.37	−1	0	+1	+2.37
*x*_1_	H_2_SO_4_ [g·L^−1^]	33.51	130.00	200.00	270.00	366.49
*x*_2_	C_2_H_2_O_4_ [g·L^−1^]	1.49	7.00	11.00	15.00	20.51
*x*_3_	Al_2_O_3_ [g·L^−1^]	0.18	5.00	8.50	12.00	16.82
*x*_4_	*T* [°C]	−1.78	12.00	22.00	32.00	45.78
*x*_5_	*t* [min]	6.22	20.00	30.00	40.00	53.78
*x*_6_	*U* [V]	5.24	8.00	10.00	12.00	14.76

**Table 2:** Significant statistical indicators for compiled mathematical models.

	1 A·dm^−2^	3 A·dm^−2^
SSE	87.51	60.30
RMSE	1.90	1.31
*R* ^2^	0.93	0.96
*R*	0.97	0.98
se	1.38	1.15
s^2^e	1.90	1.32
max⁡*e*	6.6	5.64

**Table 3:** Differences between measured layer thicknesses for current densities 1 A·dm^−2^ and 3 A·dm^−2^.

s. n.	*h*_1 A_ [*µ*m]	*h*_3 A_ [*µ*m]	Δ*h* [*µ*m]	s. n.	*h*_1 A_ [*µ*m]	*h*_3 A_ [*µ*m]	Δ*h* [*µ*m]
1	1.36	0.28	1.08	24	11.75	12.60	−0.85
2	3.91	4.06	−0.15	25	3.70	3.63	0.07
3	4.76	5.64	−0.88	26	9.91	9.59	0.32
4	7.76	7.72	0.04	27	13.55	13.70	−0.15
5	2.90	3.13	−0.23	28	13.20	12.88	0.32
6	0.63	0.95	−0.32	29	5.25	5.45	−0.20
7	2.90	3.19	−0.29	30	5.68	6.00	−0.33
8	10.78	11.00	−0.22	31	5.89	5.56	0.33
9	1.97	1.79	0.18	32	11.70	11.68	0.02
10	2.71	2.63	0.08	33	17.78	17.50	0.28
11	3.77	3.82	−0.05	34	14.23	14.45	−0.22
12	13.33	13.43	−0.10	35	3.62	1.49	2.12
13	1.79	1.27	0.52	36	6.35	7.02	−0.67
14	4.60	4.71	−0.11	37	6.21	5.74	0.47
15	7.91	9.13	−1.22	38	0.49	1.35	−0.86
16	5.74	5.35	0.39	39	0.84	0.66	0.18
17	5.12	4.63	0.49	40	14.00	10.64	3.36
18	3.78	3.43	0.35	41	1.74	2.10	−0.36
19	7.90	7.72	0.18	42	10.48	10.60	−0.12
20	15.45	18.15	−2.70	43	3.34	4.13	−0.79
21	2.14	2.62	−0.48	44	21.58	22.18	−0.60
22	9.33	9.63	−0.30	45	7.87	7.51	0.36
23	17.6	18.58	−0.98	46	7.87	7.51	0.36
